# Magnetic nanoparticle-supported eosin Y salt [SB-DABCO@eosin] as an efficient heterogeneous photocatalyst for the multi-component synthesis of chromeno[4,3-*b*]chromene in the presence of visible light

**DOI:** 10.1039/d2ra05122b

**Published:** 2022-10-11

**Authors:** Mahbube Jarrahi, Behrooz Maleki, Reza Tayebee

**Affiliations:** Department of Chemistry, School of Sciences, Hakim Sabzevari University Sabzevar 96179-76487 Iran b.maleki@umz.ac.ir; Department of Organic Chemistry, Faculty of Chemistry, University of Mazandaran Babolsar Iran

## Abstract

Heterogeneous photocatalysts present a favourable procedure to realize green and eco-friendly organic reactions. We have demonstrated an SB-DABCO@eosin catalyst in a green one-pot multi-component protocol for the production of various chromeno[4,3-*b*]chromenes *via* condensation of aromatic aldehydes and dimedone under the photo-redox catalyst bearing eosin Y using visible light. The synthesized nanocatalyst was characterized using various physicochemical techniques such as FT-IR, XRD, EDX, UV-vis, SEM, TGA and DRS. The significant advantages of the present methodology include excellent yield, cost-effectiveness, easy work-up, 100% atom economy, broad substrate scope, easy separation and efficient recycling. Furthermore, the evidence showed that the investigated condensation reaction proceeds *via* a radical mechanism, which proved the need for reactive species such as OH˙ and ˙O_2_^−^ in the photocatalytic process. In addition to the improved handling and process control, the yield of products and the rate of reactions have increased considerably in the present strategy. Reproducibility studies also guarantee good reusability and stability of the nanocatalyst for at least five runs.

## Abstract 2

Design, synthesis, and characterization of a novel magnetic nanoparticle-supported eosin Y salt as an environmentally friendly method for one-pot synthesis of chromeno[4,3-*b*]chromene in the presence of visible light.

## Graphical abstract

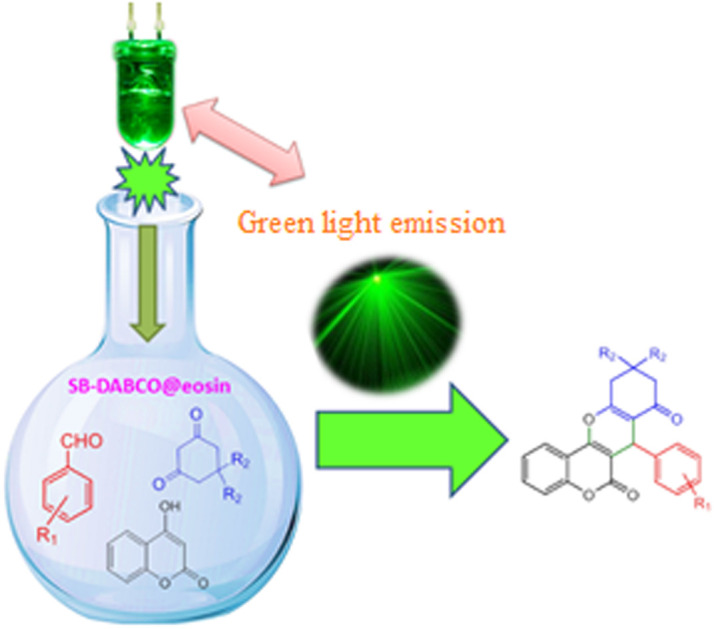

## 1. Introduction

The development of multi-component reactions (MCRs) has attracted attention in an environment friendly pathway. Multi-component reactions merge at least three reactants in a single reaction vessel to produce a product containing most atoms of the starting materials in a convergent way. MCRs as an efficient tool in drug discovery fields and synthetic organic chemistry have gained preponderance over customary multistep reactions.^1–6^ MCRs possess many apparent advantages over multistep processes, such as atom economy, high efficiency, reduced waste generation, quick and simple implementation, time-saving and energy economy. Simple separation and small amounts of used solvents or chromatographic materials are other advantages of MCRs.^7–9^

In the past few years, magnetic nanoparticles (MNPs) appeared as a new kind of semi-heterogeneous support for catalysts, and the intrinsic magnetic properties of the support allow functionally convenient separation using an external magnet. Magnetic nanoparticles are important catalysts for the synthesis of various compounds because they have a considerable advantages such as non toxicity, enhanced water solubility, elevated stability and reduced particle aggregation and reusability compared to other catalysts.^10–16^ Among different types of nanoparticles, iron oxide nanoparticles have been widely explored by researchers because they do not retain any magnetization when the magnetic field is removed. In fact, iron oxide nanoparticles (Fe_3_O_4_) have been widely applied due to their easy functionalization with polymers and other materials.^17,18^

The chromenes that are crucial oxygenated heterocyclic compounds mostly come up as an important structural component in biologically active natural compounds such as anthocyanins, alkaloids, tocopherols and flavonoids. In addition, chromene compounds have played a remarkable role in the synthesis of favorable compounds in the fields of pharmacology and drug chemistry.^19–21^ These compounds exhibit diverse biological activities and act as anti-cancer,^22^ anti-tumor,^23^ anti-HIV,^24^ anti-Alzheimer,^25^ anti-bacterial,^26^ anti-leishmanial,^27^ antitubercular,^28^ antihypertensive,^29^ antioxidative,^30^ and anti-inflammatory^31^ agents. Chromanes have been shown to have the ability to activate potassium channels and impede phosphodiesterase IV and dihydrofolate reductases and have been employed as colored optical lasers, pigments, fluorescence markers and potential biodegradable agrochemicals.^32–35^ 4*H*-Chromenes can develop the apoptotic procedure in the cancerous cells by binding to Bcl-2 anti-apoptotic proteins and therefore show anti-cancer activity.^36–39^

**Scheme 1:**
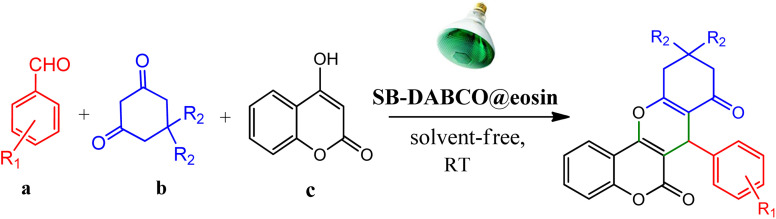
Multi-component reaction of aldehydes (a), cyclic 1,3-dicarbonyl (b) and 4-hydroxycoumarin (c) promoted by SB-DABCO@eosin.

However, in recent years, owing to the increasing attention on the organic synthesis under green chemistry, chemists redesign the synthesis methods by focusing on the visible light. Visible light has the potential to serve as an inexpensive, ubiquitous, sustainable, plentiful, unlimited, universally available source of energy as well as a nonpolluting agent for chemical reactions.^40–43^ However, most organic molecules cannot absorb visible wavelengths and this is one of the major obstacles in the development of photochemical processes which prompts the growth of efficient visible light photocatalysts in organic synthesis. Among the metal-free organic dyes, EY has been used as a profitable and environmentally friendly alternative in the structure of many photoreduction catalysts.^44^ In the mechanism for the photodegradation of organic dyes, upon irradiation with incident photons, electrons are excited to the conduction band (CB) of the photocatalyst, while holes are formed in the valence band (VB).^45–47^ Since many organic photocatalysts (and among that eosin Y) change their photophysical properties under the influence of acid–base equilibria, determining the actual nature of the dye used in the reaction conditions is of fundamental importance. It is able to transfer the activation energy to the reactants in chemical reactions.^48^ Due to its ability to absorb green light, various applications have emerged for EY as an organic photosensitizer in visible light-promoted organic synthesis.^49,50^ In continuation, we introduce a simple, more efficient, visible light promoted procedure for the synthesis of different substituted chromeno[4,3-*b*]chromene derivatives *via* combination of aryl aldehydes, 4-hydroxy coumarin and 1,3-cyclohexanedione in the presence of SB-DABCO@eosin as a green heterogeneous nanocatalyst under solventless conditions (Scheme 1). The present strategy promotes the cyclization of chromeno[4,3-*b*] chromenes *via* the formation of C–C and C–O bonds to obtain the products with excellent yield during a simple one-pot operation under mild reaction conditions. The extended procedure is an affordable alternative to devices involving metal base catalysts. Its atom economy, mild conditions, high convergence, near stoichiometric use of reactants without any additives and concomitant step economy in combination with their general compatibility rank it among sustainable synthetic methods.

## 2. Experimental section

### 2.1. Materials and methods

All materials and solvents were purchased from Merck, Sigma-Aldrich, and Fluka and were employed as received without further purification. Fourier transform infrared (FT-IR) was performed in the range 400 to 4000 cm^−1^ (PerkinElmer) using the KBr disc technique. Morphologies of the synthesized nanomaterials were analyzed using a Quantum 2000 field emission scanning electron microscope (FESEM). ^1^H- and ^13^C-NMR spectra were recorded on a 300 MHz Bruker AVANCE III spectrometer using TMS as the internal reference. A Photonix UV-visible array spectrophotometer was used to record UV-visible spectra. Elemental analyses were carried out using a Thermo Finnigan (Flash-1112EA) microanalyzer. Energy dispersive X-ray spectroscopy (EDS, Bruker QUANTAX 200) was run with an electron probe microanalyser (JEOL JXA-8230) equipped with an energy dispersive spectrometer. Melting points were determined in open capillary tubes on Stuart BI Branstead Electrothermal IA9200 apparatus. X-ray diffraction (XRD) patterns were acquired at a scanning rate of 3° min^−1^ using a Philips PW1730 diffractometer at 40 keV and 30 mA for monochromatized Cu Kα radiation in the 2*θ* domain from 10° to 80°. Thermogravimetric analyses were performed with a TGA 92 Setaram with a heating rate of 10 °C per minute.

### 2.2. General method for the synthesis of the Fe_3_O_4_ nanoparticles

Fe_3_O_4_ magnetic nanoparticles (MNPs) were synthesized according to a previous literature report with some modification. A mixture of 2.25 g FeCl_3_·6H_2_O and 0.9 g FeCl_2_·4H_2_O was dissolved in deionized water (40 ml) in a 200 ml flask. The solution was stirred vigorously (800 rpm) at RT for 10 min. Then, 7 ml of solution of 25% (w/w) ammonia was added into the mixture in a drop-wise manner over a 3 min period by means of a dropping funnel. The reaction system turned black quickly. The mixture of magnetite nanoparticles was stirred for 4 h at RT. The supernatant was decanted and magnetic Fe_3_O_4_ nanoparticles were collected by means of an external magnet and rinsed exhaustively with distilled water (20 ml) and ethanol (20 ml) until the pH of the solution reached 7. The washing operation was continued until the pH reached 7. Finally, the black MNPs were washed with 20 ml of acetone and dried in an oven at 50 °C.^51^

### 2.3. Synthesis of the Fe_3_O_4_·SiO_2_ nanoparticles (SMNPs)

0.25 g of Fe_3_O_4_ was added to a 100 ml balloon containing 40 ml of deionized water and 10 ml of 96% ethanol, and the contents of the balloon were sonicated for 30 min or more to disperse the nanoparticles well. We continued the irradiation process until we observed the dispersion of the nanoparticles well. Then 7 ml of ammonia was added dropwise to the contents of the 100 ml balloon in one min. This operation was performed under ultrasound radiation. In this case, it was exposed to ultrasound again for another 10 min. Then tetraethyl orthosilicate (TEOS, 4 ml) was added to the solution drop by drop under ultrasonic irradiation for 2 min. After adding all the TEOS to the 100 ml balloon, it was irradiated for another 5 min. So for all the work steps, the 100 ml balloon was inside the ultrasonic bath. After this, we took the balloon out of the ultrasonic bath and rotated it with a magnetic stirrer for 24 h at room temperature. Then, we washed and dried the sample as described for Fe_3_O_4_.^52^

### 2.4. Synthesis of Fe_3_O_4_@SiO_2_@Pr–Cl (SMNPs@Pr–Cl)

0.8 g SMNPs was added to a 50 ml balloon containing 30 ml of dried toluene and the contents of the balloon were sonicated for 30 min or more to disperse the fine nanoparticles. We continued the irradiation process until we observed the dispersion of the nanoparticles well. Then 4 ml of 3-chloropropyl trimethoxysilane was added to the 50 ml balloon by drop syringe over 2 min and allowed to disperse for another 10 min. The contents of the balloon were then removed from the ultrasonic water bath and rotated at 110 °C using a magnetic stirrer for 48 h under reflux conditions. The nanoparticles were then removed using a magnet and first washed with 10 ml of ordinary toluene, and then washed with deionized water and then ethanol, as in the above method. Finally, they were dried in an oven at a temperature of 50 °C for 4 to 5 h.^53^

### 2.5. Preparation of silica bonded 1,4-diazabicyclo[2.2.2]octane (SB-DABCO)

30 ml dry acetone was poured into a round-bottomed flask equipped with a refluxing condenser and 0.56 g DABCO (5 mmol) and 1 g SMNPs@Pr–Cl were added and the mixture was refluxed. After 36 h the reaction mixture was cooled at room temperature and dried under vacuum on a glass filter, and then washed with acetone, ethanol, and methanol in turn. The product obtained from this step was dried for 5 h under vacuum at 50 °C to give SB-DABCO as a white powder.^54^

### 2.6. Surface modification of SB-DABCO nanoparticles with eosin

**Scheme 2:**
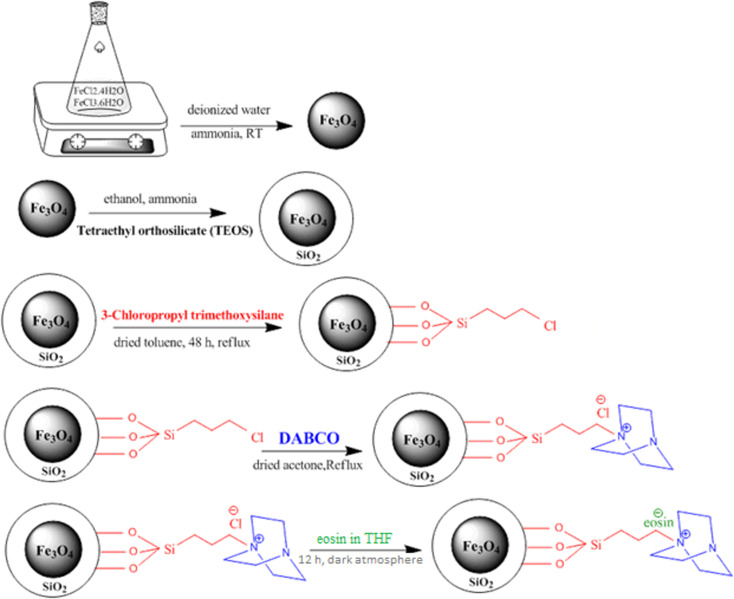
Schematic illustration of the synthesis of SB-DABCO@eosin NPs.

The SB-DABCO@eosin nanohybrid material was obtained from the well-known wet impregnation procedure. Hence, 0.02 ml eosin was dissolved in THF. Then, nano SB-DABCO (0.5 g) was slowly added to the obtained solution for 12 h under a dark atmosphere. The resulting powder was dried at 80 °C under air for 24 h. All steps of the SB-DABCO@eosin synthesis are summarized in Scheme 2.

### 2.7. General procedure for synthesis of substituted chromeno[4,3-*b*]chromene

1 mg SB-DABCO@eosin nanoparticles, 100 mg benzaldehyde (1 mmol), 162 mg 4-hydroxycoumarin (1 mmol) and 140 mg dimedone (1 mmol) were added to a 25 ml round bottom flask. The mixture was irradiated with a green light-emitting diode (*λ*_max_ = 535 nm) under open air conditions at room temperature. After completion of the reaction (controlled by TLC, *n*-hexane : ethyl acetate, 3 : 1), 3 ml H_2_O was added to the mixture and the desired chromene product was dissolved in EtOAc. After extracting the aqueous fraction with ethyl acetate (3 × 3 ml), the obtained organic layer was dried over sodium sulfate and concentrated *in vacuo*. The raw accomplished product was purified through column chromatography (100–200 mesh silica gel; ethyl acetate/hexane) to supply the pure chromene. The product was confirmed by ^1^H and ^13^C NMR spectra.

### 2.8. Spectral NMR data selected of the prepared chromenes

#### 2.8.1 Table 5, entry 1

^1^H NMR (300 MHz, DMSO-d_6_); *δ*: 7.79 (1H, d, *J* = 7.8 Hz), 7.50 (1H, d, *J* = 7.2 Hz), 7.02–7.32 (7H, m), 4.65 (1H, s), 2.63 (2H, s), 2.21 (1H, d, *J* = 15.9 Hz), 2.09 (1H, d, *J* = 16.2 Hz), 1.04 (3H, s), 0.93 (3H, s) ppm; ^13^C NMR (75 MHz, DMSO-d_6_); *δ*: 196.2, 162.1, 159.5, 153.5, 152.2, 142.4, 133.1, 129.0, 128.0, 126.8, 124.2, 122.3, 116.1, 114.2, 113.4, 105.8, 51.5, 33.0, 31.9, 28.6, 26.5 ppm.

#### 2.8.2 Table 5, entry 2

^1^H NMR (300 MHz, DMSO-d_6_) *δ* (ppm): 8.05 (2H, d, *J* = 8.7 Hz), 7.87 (1H, s), 7.86 (1H, d, *J* = 7.8 Hz), 7.52 (1H, d, *J* = 7.2 Hz), 7.44 (1H, d, *J* = 8.7 Hz), 7.35 (1H, t, *J* = 7.5 Hz), 7.29 (1H, d, *J* = 8.1 Hz), 4.80 (1H, s), 2.68 (2H, s), 2.25 (1H, d, *J* = 16.2 Hz), 2.14 (1H, d, *J* = 16.2 Hz), 1.09 (3H, s), 0.97 (3H, s); ^13^C NMR (75 MHz, MSO-d_6_) *δ* (ppm): 195.5, 163.3, 160.7, 154.8, 152.7, 150.5, 147.0, 133.4, 129.8, 124.5, 123.8, 123.3, 117.2, 13.9, 113.6, 105.3, 50.7, 33.4, 32.6, 28.5, 27.8.

#### 2.8.3 Table 5, entry 4

^1^H NMR (300 MHz, DMSO-d_6_) *δ* (ppm): 7.85–7.89 (1H, m), 7.50–7.56 (1H, m), 7.29–7.40 (2H, m), 7.15 (2H, d, *J* = 9.0 Hz), 7.09 (1H, d, *J* = 9.0 Hz), 6.66–6.73 (1H, m), 4.70 (1H, s), 3.69 (3H, s), 2.72 (2H, s), 2.21 (1H, d, *J* = 16.2 Hz), 2.09 (1H, d, *J* = 16.2 Hz), 1.12 (3H, s), 0.99 (3H, s); ^13^C NMR (75 MHz, DMSO-d_6_) *δ* (ppm): 196.3, 161.9, 160.8, 158.8, 154.0, 152.9, 136.0, 133.0, 129.9, 129.5, 124.7, 123.0, 116.9, 115.7, 113.7, 113.4, 106.5, 55.5, 50.9, 32.7, 32.5, 29.5, 27.6.

#### 2.8.4 Table 5, entry 5

^1^H NMR (300 MHz, DMSO-d_6_) *δ* (ppm): 8.09 (1H, s), 7.89 (1H, d, *J* = 8.1 Hz), 7.83 (1H, d, *J* = 7.8 Hz), 7.65 (1H, d, *J* = 6.6 Hz), 7.54 (1H, t, *J* = 7.5 Hz), 7.25–7.39 (2H, m), 7.17 (1H, d, *J* = 8.1 Hz), 4.88 (1H, s), 2.70 (1H, d, *J* = 18 Hz), 2.53 (1H, d, *J* = 18 Hz), 2.15 (1H, d, *J* = 16.5 Hz), 2.12 (1H, d, *J* = 16.5 Hz), 1.05 (3H, s), 0.99 (3H, s); ^13^C NMR (75 MHz, DMSO-d_6_) *δ* (ppm): 196.2, 163.3, 159.6, 154.0, 152.2, 147.9, 145.0, 135.4, 133.0, 129.7, 124.9, 123.5, 123.3, 121.9, 117.0, 113.4, 105.0, 50.4, 39.3, 33.6, 32.3, 28.9, 27.3.

#### 2.8.5 Table 5, entry 6

^1^H NMR (300 MHz, DMSO-d_6_) *δ* (ppm): 7.97–8.03 (2H, m), 7.88–7.90 (2H, m), 7.57–7.61 (1H, m), 7.29–7.51 (3H, m), 5.10 (1H, s), 2.83–2.99 (2H, m), 2.43–2.49 (2H, m), 2.09–2.23 (2H, m); ^13^C NMR (75 MHz, DMSO-d_6_) *δ* (ppm): 196.3, 164.9, 153.0, 145.0, 136.3, 132.6, 129.1, 124.2, 123.0, 122.9, 122.4, 117.3, 115.5, 113.6, 105.5, 36.9, 33.8, 27.5, 20.4.

#### 2.8.6 Table 5, entry 7

^1^H NMR (300 MHz, DMSO-d_6_) *δ* (ppm): 8.08 (1H, d, *J* = 7.8 Hz), 7.79 (1H, t, *J* = 7.5 Hz), 7.49–7.59 (2H, m), 7.37 (2H, d, *J* = 8.4 Hz), 6.95 (2H, d, *J* = 7.8 Hz), 3.89 (3H, s), 4.94 (1H, s), 2.95–3.13 (2H, m), 2.58 (2H, t, *J* = 6.3 Hz), 2.19–2.33 (2H, m); ^13^C NMR (75 MHz, DMSO-d_6_) *δ* (ppm): 195.9, 163.9, 160.8, 159.9, 153.9, 153.0, 135.9, 133.2, 130.2, 125.2, 123.4, 121.2, 116.9, 116.5, 114.0, 113.5, 112.0, 106.9, 55.6, 37.2, 32.9, 27.0, 20.5.

## 3. Results and discussion

In this paper, we have tried to synthesize substituted chromeno[4,3-*b*]chromenes using SB-DABCO@eosin NPs. Some procedures have been reported for the production of chromeno[4,3-*b*]chromene derivatives *via* combination of arylaldehydes with 4-hydroxycoumarine and cyclic-1,3-diketone compounds in the presence of *p*-TSA, Fe[DS]_3_, [DMDBS]·2HSO_4_, and I_2_/HOAc.^54–57^ However, most of these techniques bear at least one of the following disadvantages: difficult and boring work-up procedures, long reaction time, high operating temperature, non-recyclable or expensive metal catalysts, moderate yields and poor selectivity, commercially unavailable compounds, corrosive and harmful organic solvents, and limited substrate scope.

Therefore, it seems that the main work is to replace inefficient methods with more plausible methods based on recyclable, stable, and improved catalysts. Environmental and economic concerns have turned researchers' attention to green chemical processes in organic synthesis. In recent years, photochemical techniques, especially using visible light, have become an efficient tool for chemical reactions. The superiority of using a visible light photo redox catalyst is its ability to activate atmospheric oxygen, a natural green oxidizing agent in visible light, controlled by organic transformations involving single electron transfer (SET). Among the organic dyes of the fluorescein family, the organic dye eosin Y has found a better place in photocatalyzed organic reactions due to its better performance capacity.^48^

### 3.1. Characterization and physicochemical properties of [SB-DABCO@eosin] nanoparticles

The synthesized supported [SB-DABCO@eosin] nanocatalyst was characterized by means of XRD, UV-vis, DRS, FT-IR, SEM, TGA and EDX. The surface interaction between eosin and [SB-DABCO@eosin] can be investigated by using FT-IR and UV-vis analyses. The material was analyzed by different analytical and spectroscopic techniques. The XRD patterns of the materials reported the formation of nano sized particles. The morphology and particle size of the [SB-DABCO@eosin] NPs were further confirmed by scanning electron microscopic (SEM) study. Energy dispersive X-ray analysis through SEM obviously showed the high purity of the sample.

#### 3.1.1 XRD analyses

**Fig. 1:**
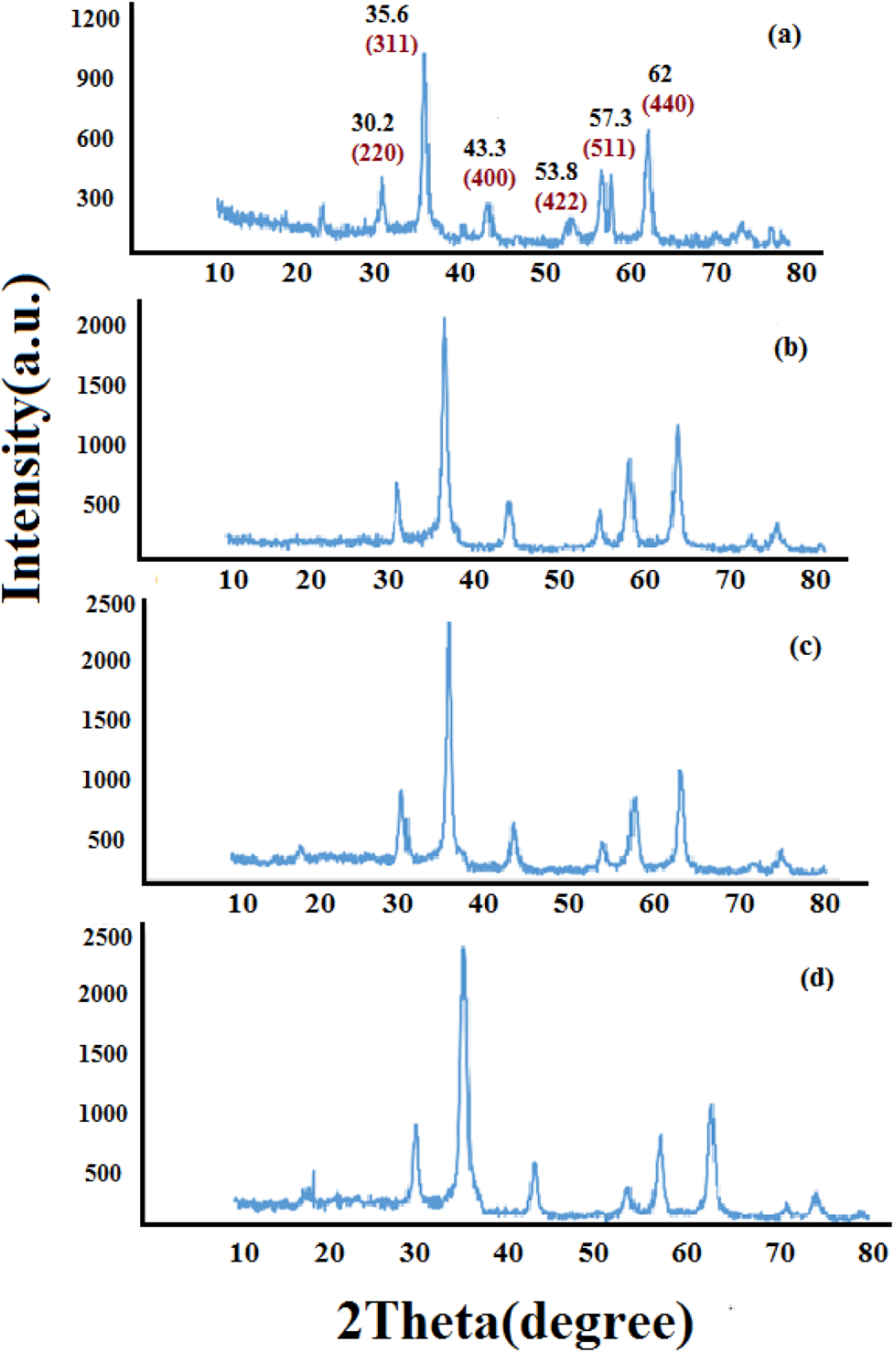
Experimental XRD patterns for the synthesized Fe_3_O_4_ (a), SMNPs (b), and SMNPs@DABCO (c) compared with the simulated SMNPs@DABCO@eosin (d).

Fig. 1 shows the XRD pattern of synthesized nanomaterials Fe_3_O_4_ (MNPs), SMNPs, SMNPs@DABCO and SMNPs@DABCO@eosin. The positions and relative intensities of all diffraction peaks in the XRD pattern of MNPs matched with the reference pattern of the cubic inverse spinel structure of magnetite (JCPDS card number 19-0629). Six sharp Bragg diffraction peaks at the 2*θ* of 30.2°, 35.6°, 43.3°, 53.8°, 57.3°, and 62° are the corresponding peaks of (220), (311), (400), (422), (511), and (440) miller indices, respectively (Fig. 1a). Repetition of the peaks mentioned above, without any additional peaks, in the XRD patterns of the products of different stages of the catalyst synthesis indicates the preservation of the magnetite structure in the nanoparticles even after the surface modification.^58^ However, there was no detectable diffraction, indicating the presence of silica, which could be attributed to its high dispersity over the nanocomposites. The presence of DABCO does not have a significant effect on the cubic spinel crystal structure of Fe_3_O_4_ nanoparticles and as a result in their XRD pattern, again, it shows no phase change after surface modification of magnetite nanoparticles. Moreover, the size of crystalline magnetite nanoparticles can be calculated by using the XRD data in the Debye–Scherrer formula (*D* = *kλ*/*β* cos *θ*, *λ* = 0.15406 nm); here, the peak of the highest intensity (311, 2*θ* = 35.6) was chosen to define the diameter of the nanoparticles. Thus, the size of SMNPs@DABCO@eosin particles was attained to be ∼80 nm, in accord with the FESEM analysis.

#### 3.1.2 UV-vis and DRS analysis

**Fig. 2:**
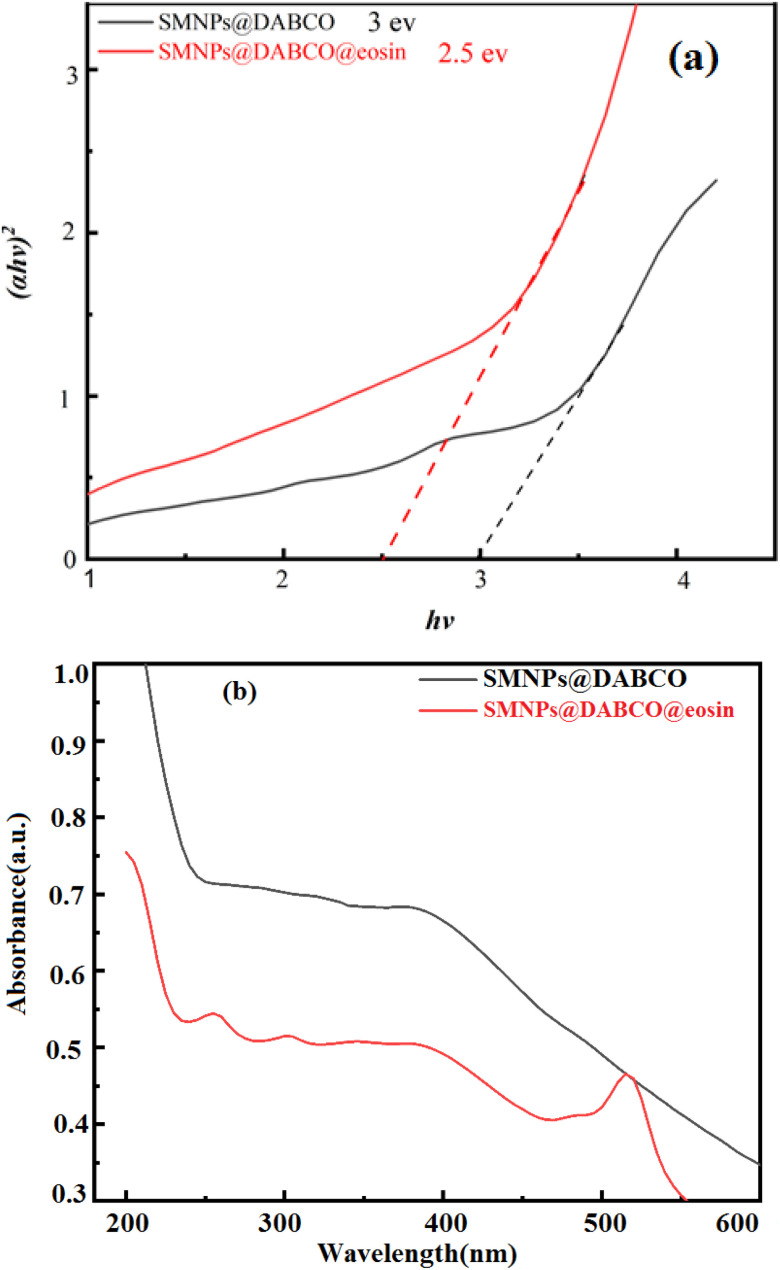
(a) DRS of SMNPs@DABCO and SMNPs@DABCO@eosin and (b) UV-vis absorption spectrum.

The UV-vis absorption spectra of SMNPs@DABCO and SMNPs@DABCO@eosin were studied with the purpose of investigating the mechanism involved in the efficient visible light absorption. Diffuse reflectance spectra were recorded to estimate the observed band-gap of the catalyst. Estimation of the optical band gaps of samples to photon energy was done using the extrapolation of the linear slope of the Tauc plot (Fig. 2). The calculated band-gap energy of SMNPs@DABCO and SMNPs@DABCO@eosin was found to be 3 and 2.5 eV, respectively. To obtain the optical energy band gap (*E*_g_), the extrapolation plot of *hν versus* (*αhν*)^2^ was used (Fig. 2b). In this relation, the analysis is carried out based on the equation *αhν* = *A*(*hν* − *E*_g_)_*n*_, where *A* is a constant and *α*, *hν*, and *E*_g_ refer to the absorption coefficient, light frequency and band-gap, respectively.^59^ We think that eosin reduces the optical band gap and, therefore, reduction of absorbance. In this case, photons cannot excite electrons from the valence band (VB) to the conduction band (CB). In addition, functionalization of catalyst nanoparticles with eosin affects the electronic features of surface electrons.^60–62^

#### 3.1.3 FT-IR spectroscopy

**Fig. 3:**
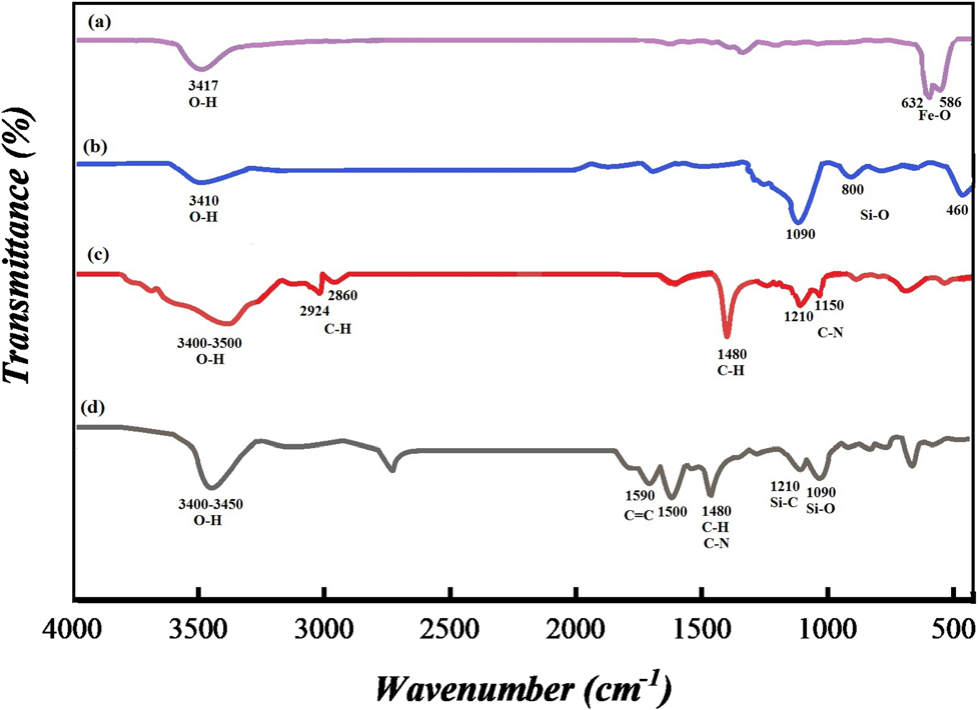
FT-IR spectra of (a) Fe_3_O_4_, (b) SMNPs, (c) SMNPs@DABCO and (d) SMNPs@DABCO@eosin.

FT-IR spectroscopy was applied to recognize the successful synthesis of SMNPs@DABCO@eosin. Hence all fragments including (a) Fe_3_O_4_, (b) SMNPs, (c) SMNPs@DABCO and (d) SMNPs@DABCO@eosin were studied. Intense peaks near 586 and 632 cm^−1^ correspond to Fe–O stretching vibration (Fig. 3a). In the spectra displayed in Fig. 3b, the advent of characteristic peaks at 1090, 800 and 460 cm^−1^ is related to Si–O–Si stretching, Si–O bending, and Si–O–Si bending vibrations respectively. The anchoring of DABCO on the SMNP fragment is confirmed by the appearance of symmetric and asymmetric CH_2_ of the alkyl chains at 2860 and 2924 cm^−1^, CH_2_ sharp bending peak at 1480 cm^−1^ and C–N stretching peaks at 1150 and 1210 cm^−1^ (Fig. 3c).^58,63,64^ In Fig. 3d, the presence of absorption bands at 1590 and 1500 cm^−1^, which are related to the carboxyl functional group and C=C of aromatic phenyl rings, respectively, confirms the loading of EY on the SMNP@DABCO surface.^65^

#### 3.1.4 FESEM and EDS of SMNPs@DABCO@eosin

**Fig. 4:**
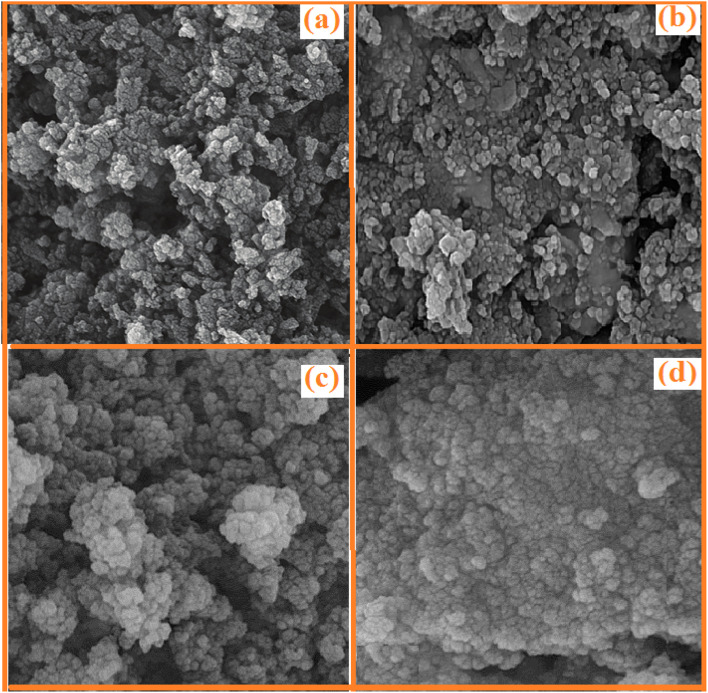
The SEM images of SMNPs (a), SMNPs@Pr–Cl (b), SB-DABCO (c) and SMNPs@DABCO@eosin (d).

**Fig. 5:**
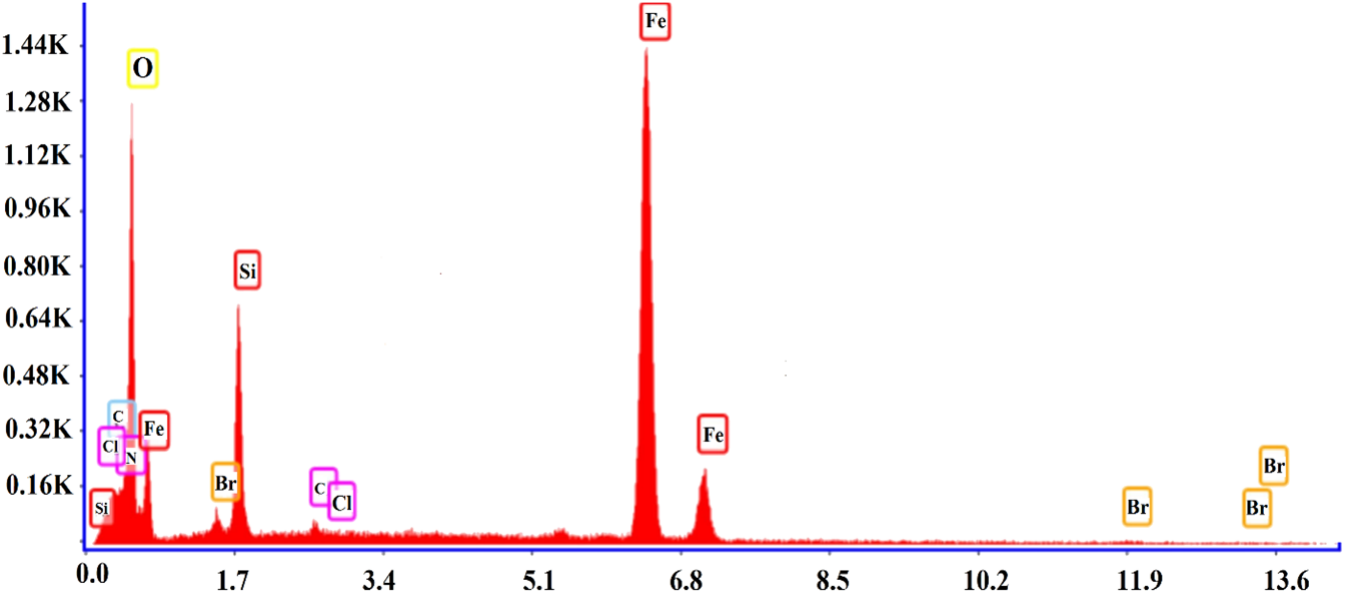
The EDS analysis of SMNPs@DABCO@eosin.

FESEM is an efficient method to characterize the morphological features of the synthesized structure. The surface morphologies of SMNPs, SMNPs@Pr–Cl, SB-DABCO and SMNPs@DABCO@eosin are provided in Fig. 4a–d. By consideration of the SEM image, it can be derived that the synthesized nanocatalyst has almost a nanosphere framework and the diameter of these nanospheres was less than 100 nm. EDS analysis confirmed the symbiosis of Fe, Si and O in the nanocomposite. Energy-dispersive X-ray spectroscopy is often utilized to present beneficial data about the elemental composition of the compounds. According to Fig. 5, it is detailed that SMNPs@DABCO@eosin possess all predicted elemental cases including C, N, O, Si, Cl, Fe, and Br in the data from the EDS coupled SEM analysis.

#### 3.1.5 TGA curve of SMNPs@DABCO@eosin

**Fig. 6:**
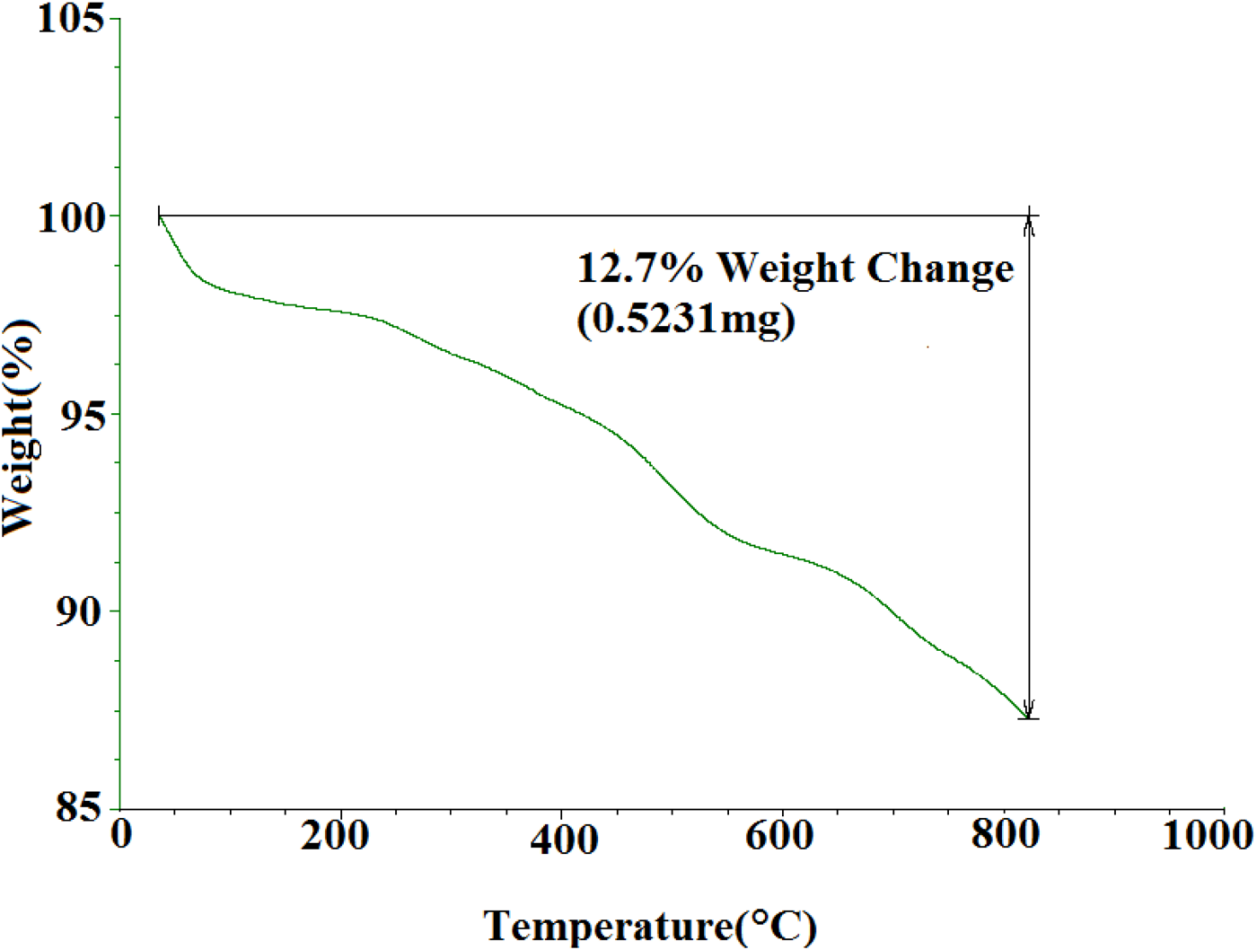
TGA profile for SMNPs@DABCO@eosin.

TGA was used to investigate the thermal stability of synthesized SMNPs@DABCO@eosin (Fig. 6). As the temperature rises, several steps of decomposition are apparent from Fig. 6. The initial weight loss observed below 200 °C is most probably caused by the water desorption from the nanoparticle surface.^66^ The EY segment was destructed at about 200–380 °C.^65^ Thermal decomposition of the 3-chloropropyl silane groups was also done between 270 and 480 °C.^67^ Research has determined that MNPs-DABCO has been degraded at 250–500 °C and the DABCO fragment is separated from MNPs.^67^ Also the latest weight loss, above 600 °C, has indicated the presence of remaining silica and Fe_3_O_4_ nanoparticles.

#### 3.1.6 VSM analysis

**Fig. 7:**
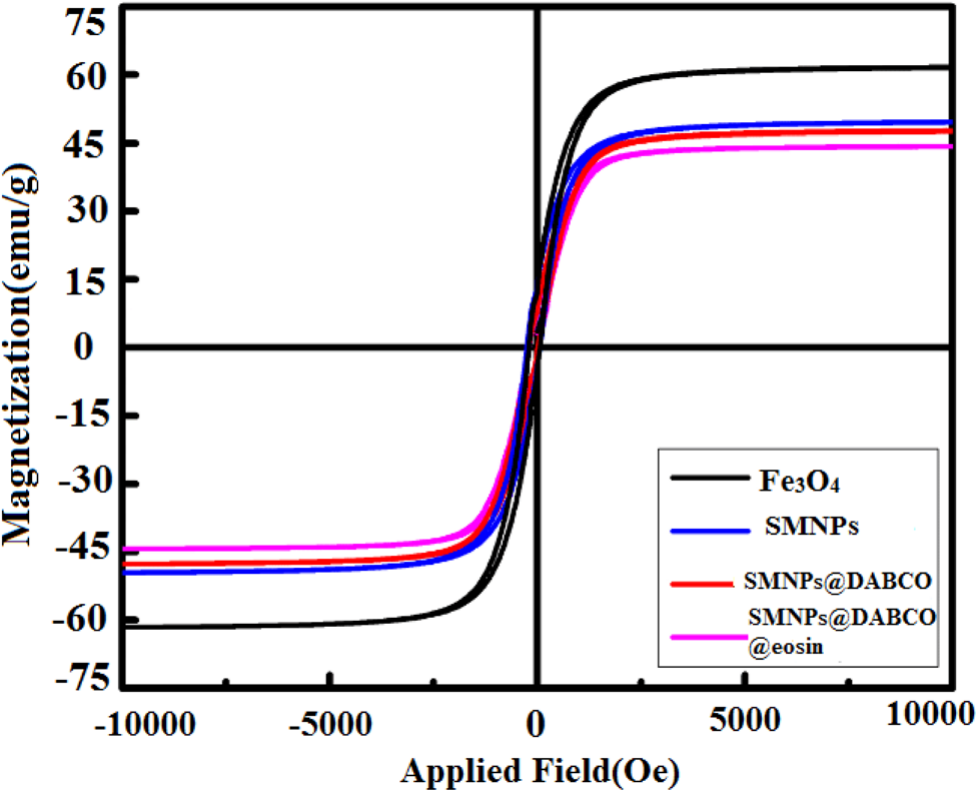
Room-temperature magnetization curves of Fe_3_O_4_, SMNP, SMNPs@DABCO, and SMNPs@DABCO@eosin.

VSM analysis was performed to display the magnetic features of SMNPs@DABCO@eosin that should be compared to those obtained for the primary core at room temperature. In Fig. 7, the hysteresis loops at room temperature are drawn to estimate the magnetic behavior of these synthesized nanocompounds. As forecast, anchoring the consecutive layers on the Fe_3_O_4_ nanoparticles drops the magnetic saturation values of Fe_3_O_4_ nanoparticles (from 60 emu g^−1^ to 41 emu g^−1^). In addition, due to the magnetic core–shell nanocomposite, the as-synthesized photocatalyst can be simply gathered from aqueous solution by an external magnetic field for a little while and then can be redispersed by lightly shaking it. The outcomes are consistent with the rising number of layers onto the primary magnetic core surface. In total, the obtained evidence proves the successful immobilization on modified Fe_3_O_4_ magnetic nanoparticles.

### 3.2. Catalytic tests

To investigate the photocatalytic activity of SMNPs@DABCO@eosin, a model reaction was designed using 1 mmol benzaldehyde, 1 mmol dimedone, and 1 mmol 4-hydroxycoumarin in the presence of the photocatalyst under irradiation with LED light. The reaction mixture was magnetically stirred in an open air test tube. To derive the best reaction conditions, various parameters of condensation reaction, such as irradiation source, amount of photocatalyst, reaction time, type of solvents and temperature, were optimized.

#### 3.2.1 Studying the effect of the light source

One of the main factors affecting the efficiency of photocatalytic reactions is the irradiation source; hence, the model reaction was subjected to different light irradiations (Table 1). The results of this survey showed that if the reaction be done under the dark and some color light LED conditions, different yields would be obtained under the same conditions and the best efficiency was obtained for green LED light irradiation (*λ* = 535 nm).

**Table 1:** Optimization of the irradiation source in condensation reaction^a^

Entry	Light source	Light specification	SMNPs@DABCO@eosin	Time (h)	Yield (%)
1	Dark	—	0.001	2	15
2	White LED	20 × 1 W	0.001	2	19
3	Blue	2.5 W, *λ* = 431 nm	0.001	2	47
4	Red	2.5 W, *λ* = 695 nm	0.001	2	44
5	Green	2.5 W, *λ* = 535 nm	0.001	2	62
6	Violet	2.5 W, *λ* = 455 nm	0.001	2	40

a Reaction conditions: benzaldehyde (1 mmol), 4-hydroxycoumarin (1 mmol), dimedone (1 mmol), SMNPs@DABCO@eosin (1 mg), solvent free, room temperature, open atmosphere.

#### 3.2.2 Studying the effect of catalyst amount

**Fig. 8:**
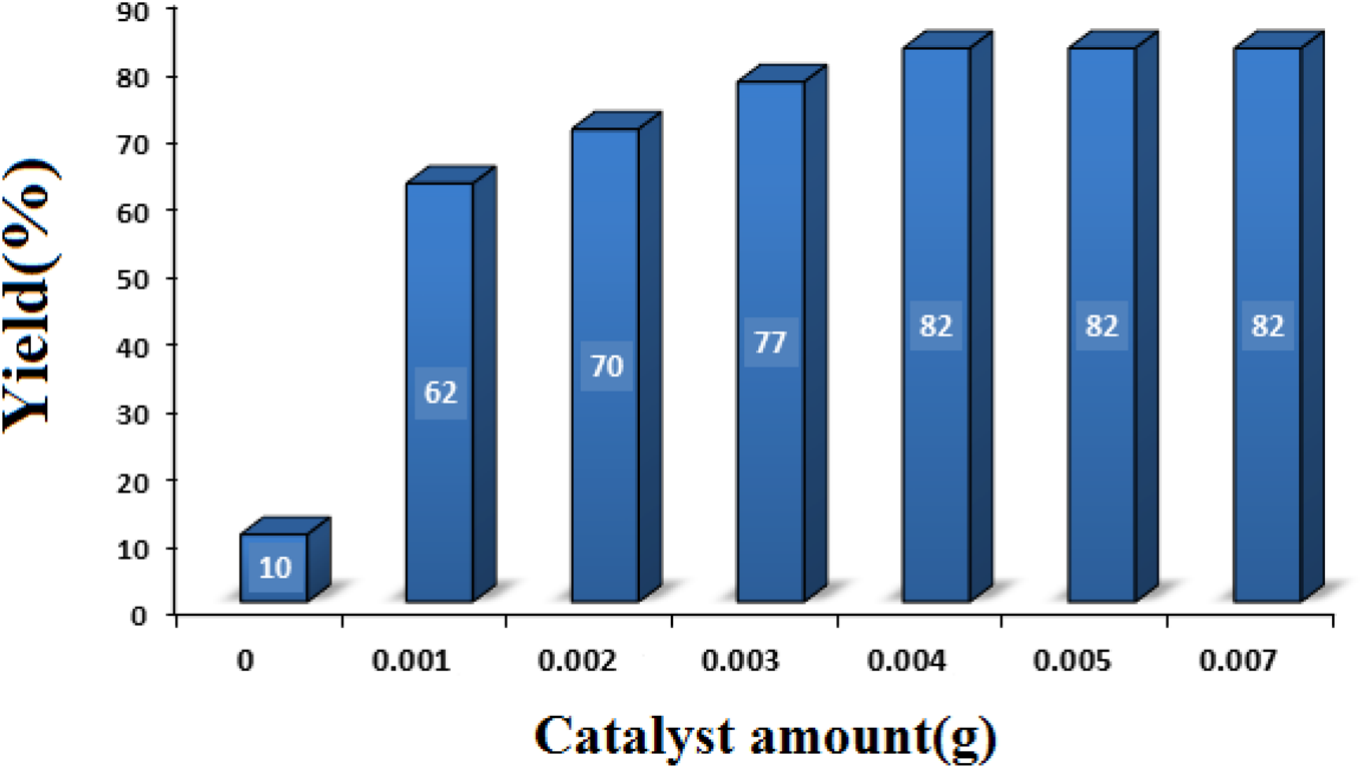
Optimization of the catalyst amount in condensation reaction [reaction conditions: benzaldehyde (1 mmol), 4-hydroxycoumarin (1 mmol), dimedone (1 mmol), solvent free, room temperature, open atmosphere, green LEDs (2.5 W, *λ* = 535 nm), time: 2 h].

With the aim of achieving the optimal amount of catalyst, the model reaction was performed in the presence of different amounts of SMNPs@DABCO@eosin (0–0.005 g). As seen in Fig. 8, 0.004 g of SMNPs@DABCO@eosin gave the best efficiency (82%) under the chosen reaction conditions. Nonetheless, extra enhancement of the catalyst amount modified neither the reaction time nor the efficiency.

#### 3.2.3 Studying the effect of reaction time

**Fig. 9:**
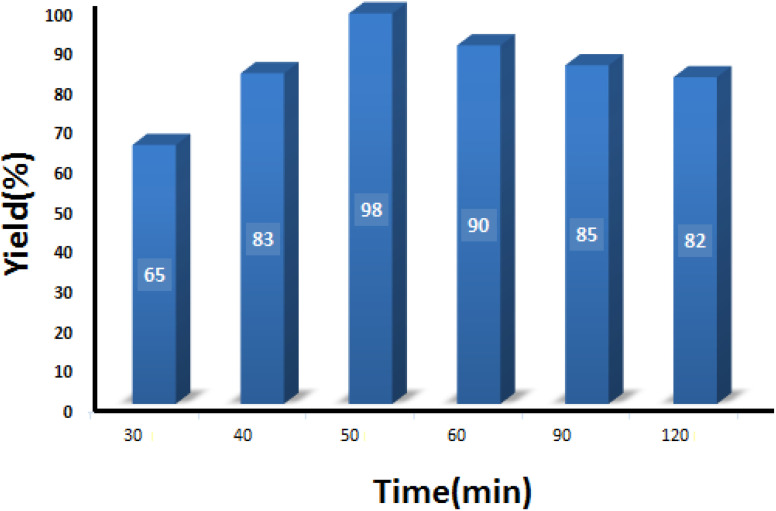
Optimization of the reaction time in condensation reaction [benzaldehyde (1 mmol), 4-hydroxycoumarin (1 mmol), dimedone (1 mmol), SMNPs@DABCO@eosin (0.004 g), solvent free, room temperature, open atmosphere, green LEDs (2.5 W, *λ* = 535 nm)].

The model reaction was conducted at different reaction times to obtain the optimum time recommended to get the maximum product. As can be seen in Fig. 9, 50 min was sufficient to attain the maximum output of 98%. It is worth noting that increasing the reaction time caused a slight decrease in the yield percentage, which can be related to the poisoning of the catalyst surface.

#### 3.2.4 Studying the effect of solvents

To optimize the reaction solvent, the condensation reaction of benzaldehyde, 4-hydroxycoumarin and dimedone was done in some chosen solvents such as H_2_O, toluene, ethanol and acetonitrile under optimum conditions. The results showed that the reaction has the best yield under solvent-free conditions (98% after 50 min, Table 2, entry 1).

**Table 2:** Optimization of solvent in condensation reaction^a^

Entry	SMNPs@DABCO@eosin (g)	Solvent	Time (min)	Yield^b^ (%)
1	0.004	—	50	98
2	0.004	H_2_O	50	86
3	0.004	Toluene	50	62
4	0.004	Ethanol	50	84
5	0.004	CH_3_CN	50	70

a Reaction conditions: benzaldehyde (1 mmol), 4-hydroxycoumarin (1 mmol), dimedone (1 mmol), SMNPs@DABCO@eosin (0.004 g), room temperature, open atmosphere, green LEDs (2.5 W, *λ* = 535 nm).

b Isolated yield of product.

#### 3.2.5 Studying the effect of temperature

To elevate the product and achieve the best reaction conditions, finally we focused on the effect of temperature on the reaction condensation of benzaldehyde with 4-hydroxycoumarin and dimedone (Table 3). As can be seen an increase in temperature didn't increase the yield and the best outcome was gained at 25 °C after 50 min; therefore, 25 °C (room temperature) was kept as the reaction temperature for all runs.

**Table 3:** Optimization of temperature in condensation reaction^a^

Entry	SMNPs@DABCO@eosin (g)	Temperature (°C)	Time (min)	Yield^b^ (%)
1	0.004	25	50	98
2	0.004	55	50	88
3	0.004	82 (reflux)	50	90

a Reaction conditions: benzaldehyde (1 mmol), 4-hydroxycoumarin (1 mmol), dimedone (1 mmol), SMNPs@DABCO@eosin (0.004 g), solvent free, open atmosphere, green LEDs (2.5 W, *λ* = 535 nm).

b Isolated yield of product.

#### 3.2.6 Role of hole and electron scavengers

**Fig. 10:**
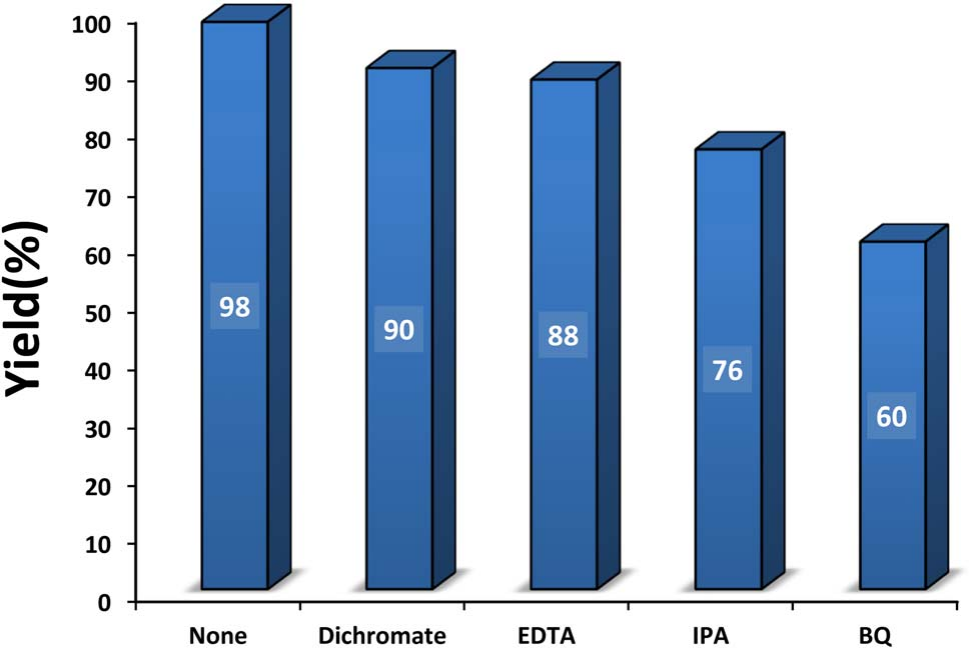
Effect of some familiar scavengers on condensation reaction [benzaldehyde (1 mmol), 4-hydroxycoumarin (1 mmol), dimedone (1 mmol), SMNPs@DABCO@eosin (0.004 g), solvent free, room temperature, open atmosphere, green LEDs (2.5 W, *λ* = 535 nm), time: 50 min].

Hole and electron scavengers were involved in the photocatalytic condensation of benzaldehyde with 4-hydroxycoumarin and dimedone in the presence of SMNPs@DABCO@eosin as a heterogeneous nanophotocatalyst (Fig. 10). Broadly, superoxide anion-radical, hydroxyl radical and holes are effective radical species in a photocatalysis reaction. Consequently, several common scavengers were investigated in the present study. In these experiments dichromate and ethylenediaminetetraacetic acid (EDTA) were employed as an electron scavenger^68^ and h^+^ scavenger,^69^ respectively. In this case, yield% was decreased 8% and 10% respectively, which confirmed probable contribution of e^−^ and h^+^ in the photocatalysis reaction. In the presence of isopropyl alcohol (IPA), as a hydroxyl free radical scavenger,^70^ yield% was reduced from 98 to 76%. In addition, a great decrease of yield% (38%) in the presence of *p*-benzoquinone (BQ) as an ˙O_2_^−^ scavenger^71^ proved that ˙O_2_^−^ would strongly affect the photocatalytic process. Overall, it can be concluded from the data that OH˙ and ˙O_2_^−^ would be the main species in the photocatalytic procedure.^72,73^

**Scheme 3:**
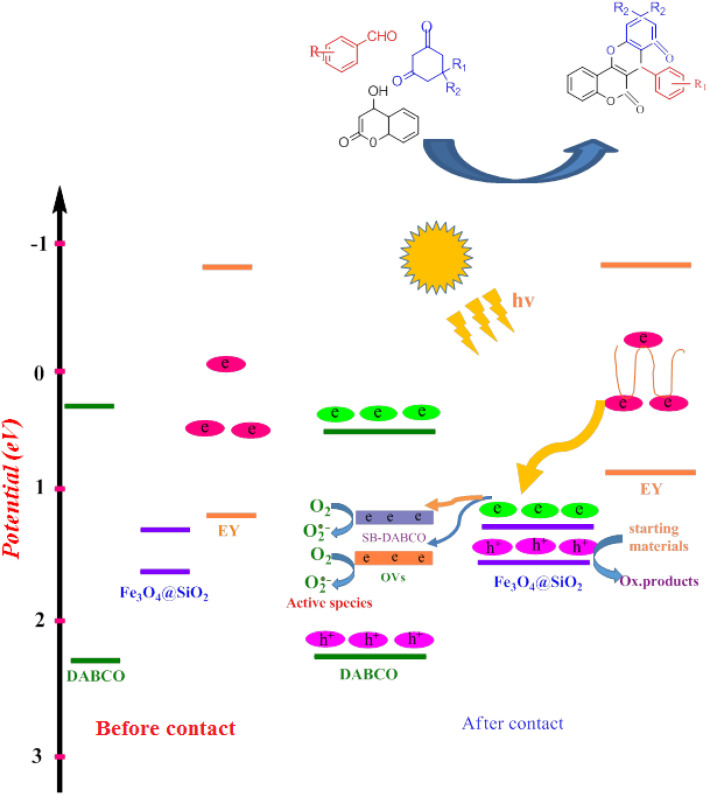
Band position of DABCO, EY, SMNPs and the produced heterojunctions.

In a common photocatalytic system, choosing an appropriate electron carrier is necessary to facilitate effective transfer of charge carriers and enhance the stability of the photocatalyst. Fe_3_O_4_·SiO_2_@DABCO (SMNPs@DABCO) and eosin fragments absorb sufficient energy to produce electron–hole pairs upon exposure to the green LEDs. The results of this study show that light absorption remarkably increased with the as-prepared hierarchical SMNPs@DABCO@eosin nanocomposite. The photoinduced electrons in SMNPs@DABCO CB tend to shift toward the interface of the heterojunction and endeavor to combine with the holes of eosin (EY) VB. Thus, SMNPs contribute to the process and bridges between the DABCO and EY, enhancing the combination rate. Therefore, the recombination rate of charge carriers drastically suppresses within SMNPs@DABCO and EY, leading to accumulation of electrons in EY CB and holes in the VB of SMNPs@DABCO. Ultimately, the accumulated electrons with strong reducibility would reduce O_2_ to ˙O_2_^−^ (Scheme 3).^74^

#### 3.2.7 Comparison of the present protocol with some reported similar methods

The gained results for the catalytic activity of SMNPs@DABCO@eosin were compared with a number of similar reports on the preparation of chromeno[4,3-*b*]chromenes in terms of catalyst amount, reaction time, temperature, solvent and yield%, as shown in Table 4. Due to the use of a very small amount of catalyst, relatively low time, low temperature, no use of solvent and high efficiency, compared to the investigated methods, the present method is a special case that provides a successful route for the one-step/one-pot synthesis of the desired products under very mild conditions.

**Table 4:** Comparison of the catalytic activity of SMNPs@DABCO@eosin with some reported catalysts in the synthesis of chromeno[4,3-*b*]chromene^a^

Catalyst	Time (min)	Temp (°C)	Solvent	Yield (%)	Ref.
SMNPs@DABCO@eosin (4 mg)	50	r.t.	—	98	This work
Eosin Y (2 mol%)	210	r.t.	CH_3_CN	77	19
Fe_3_O_4_@SiO_2_@(CH_2_)_3_OMoO_3_H (2 mg)	45	80	—	90	75
t-ZrO_2_ np (10 mol%, 12.3 mg)	30	80	H_2_O (5 ml)	91	76
CuFe_2_O_4_@SO_3_H (0.05 g)	150	70	EtOH	90	77
Fe(DS)_3_ (10 mol%)	120	70	H_2_O	87	57

a Benzaldehyde was used in all cases.

#### 3.2.8 Synthesis of different chromeno[4,3-*b*]chromenes catalyzed by SMNPs@DABCO@eosin

To define the extent, limitations and versatility of the present strategy, various aromatic aldehydes were reacted with diketone and 4-hydroxycoumarin for production of different chromeno[4,3-*b*]chromenes under solvent free conditions and green LEDs (2.5 W, *λ* = 535 nm). As mentioned in Table 5, aromatic aldehydes bearing different electron releasing/withdrawing substituents were able to create the desired product in good to excellent yield in short reaction time with superior selectivity. The products were purified by recrystallization from ethanol and the second step from ethyl acetate.^62^ The products were characterized by spectroscopic and spectrometric methods.
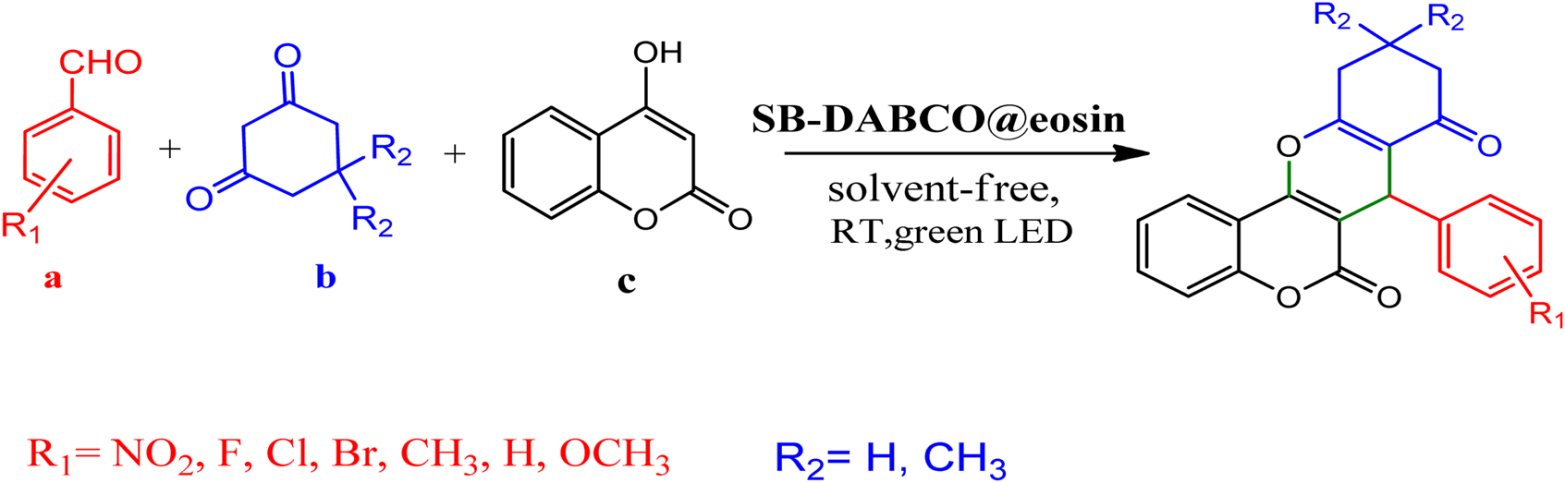


**Table 5:** Preparation of chromeno[4,3-*b*]chromene^a^

Entry	Aldehyde	Dimedone	Product	Yield (%)	M.p
					Found, reported^78–80^
1	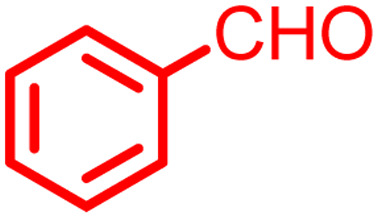	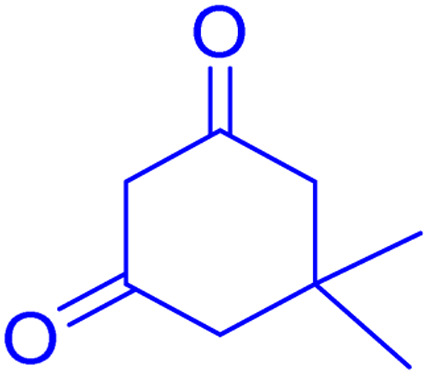	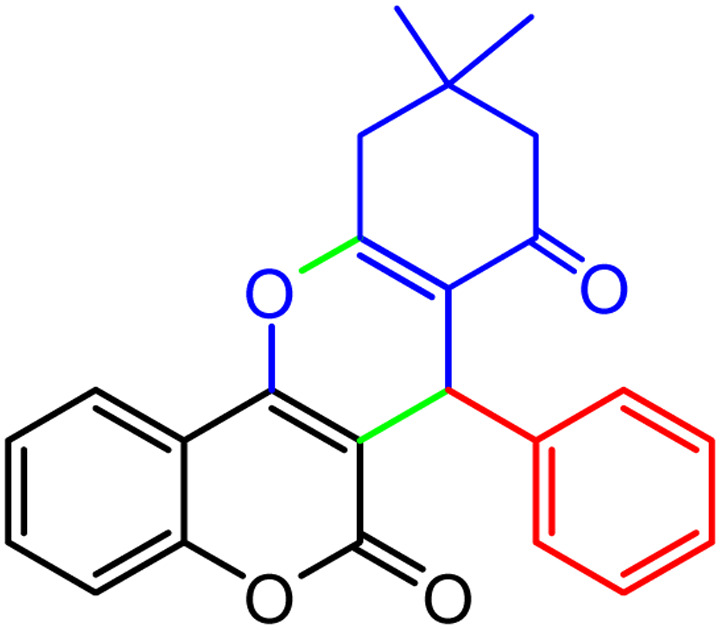	90	223–224, 220–222
2	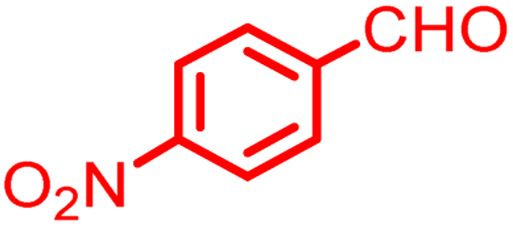	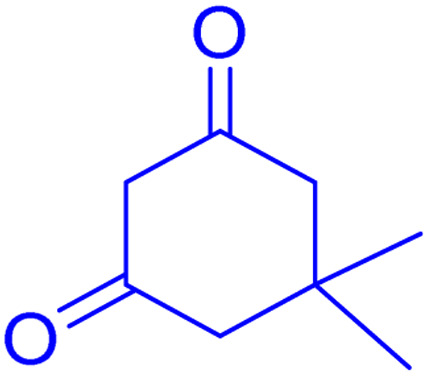	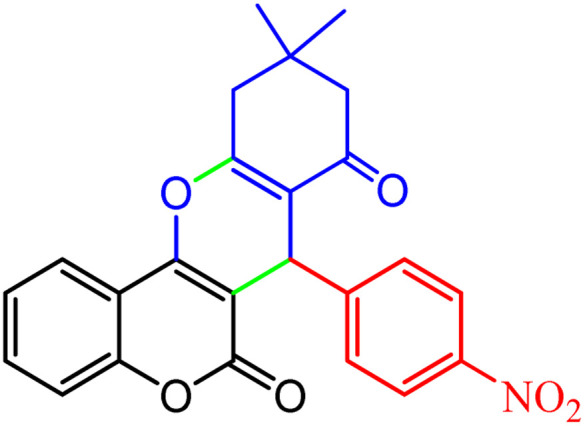	96	209–211, 208–210
3	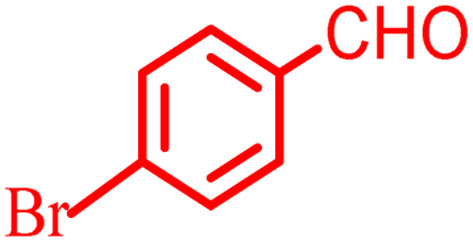	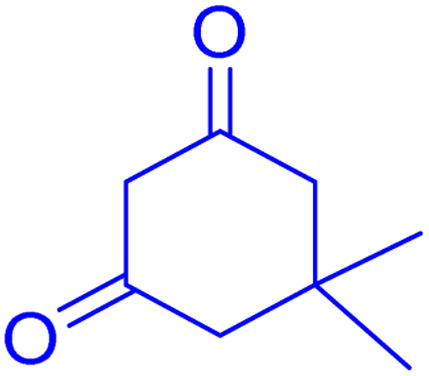	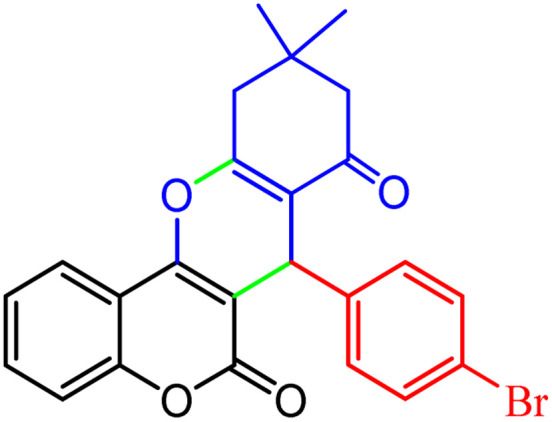	76	227–229, 228–230
4	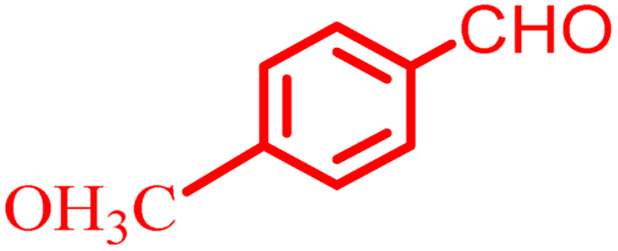	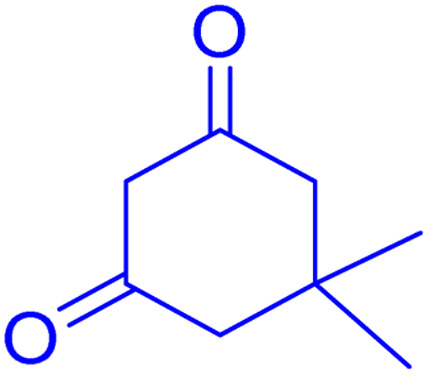	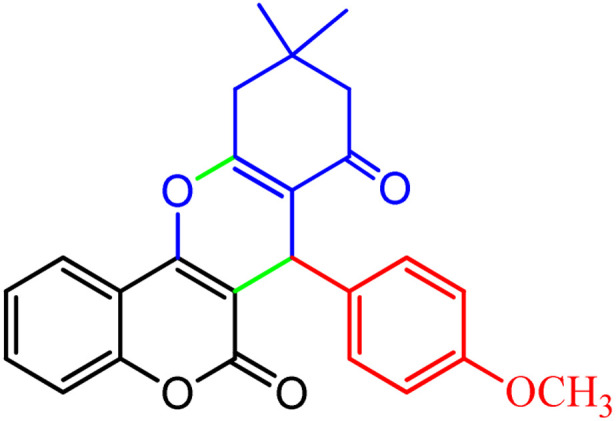	75	190–191, 187–189
5	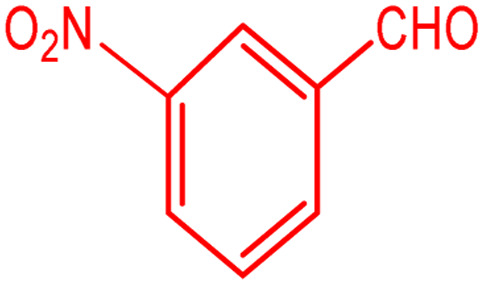	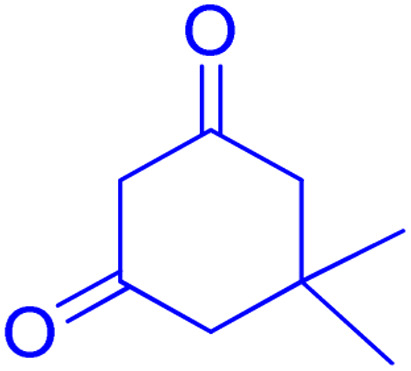	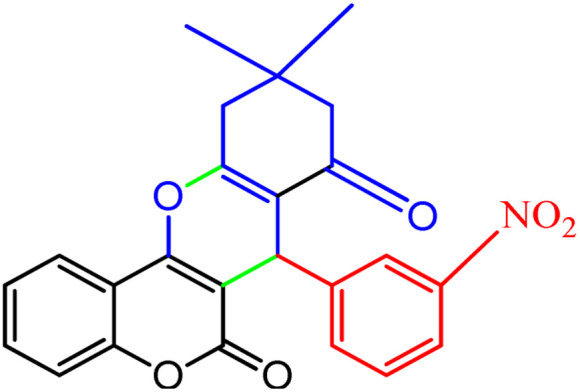	79	233–234, 230–232
6	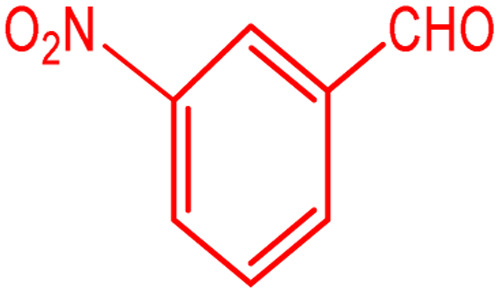	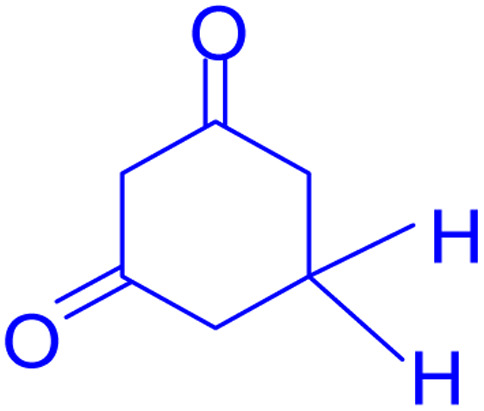	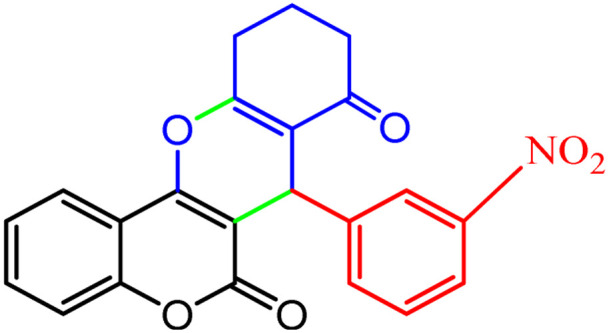	77	220–222, 218–220
7	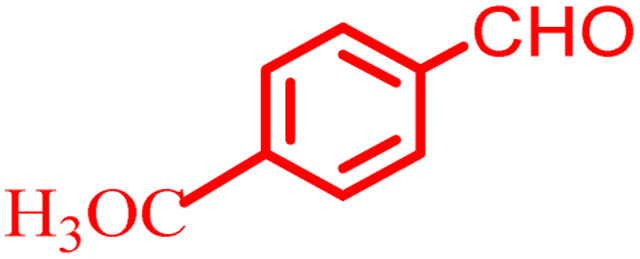	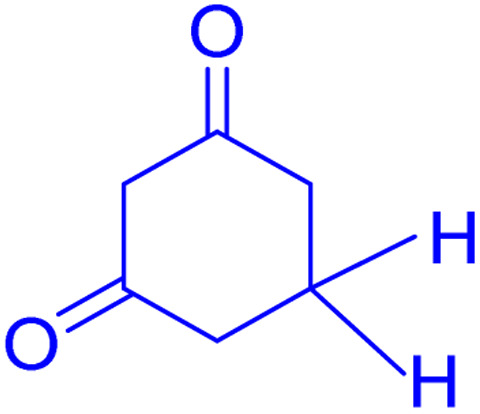	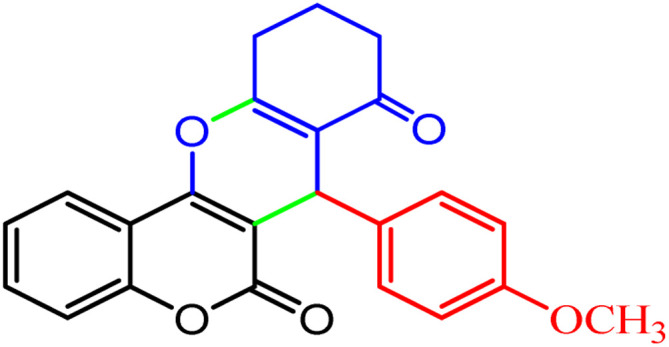	78	196–198, 196–197

a Reaction conditions: benzaldehyde (1 mmol), 4-hydroxycoumarin (1 mmol), dimedone (1 mmol), SMNPs@DABCO@eosin (0.004 g), solvent free, room temperature, open atmosphere, green LEDs (2.5 W, *λ* = 535 nm), time: 50 min.

#### 3.2.9 Studying the stability and reusability of nano SMNPs@DABCO@eosin

**Fig. 11:**
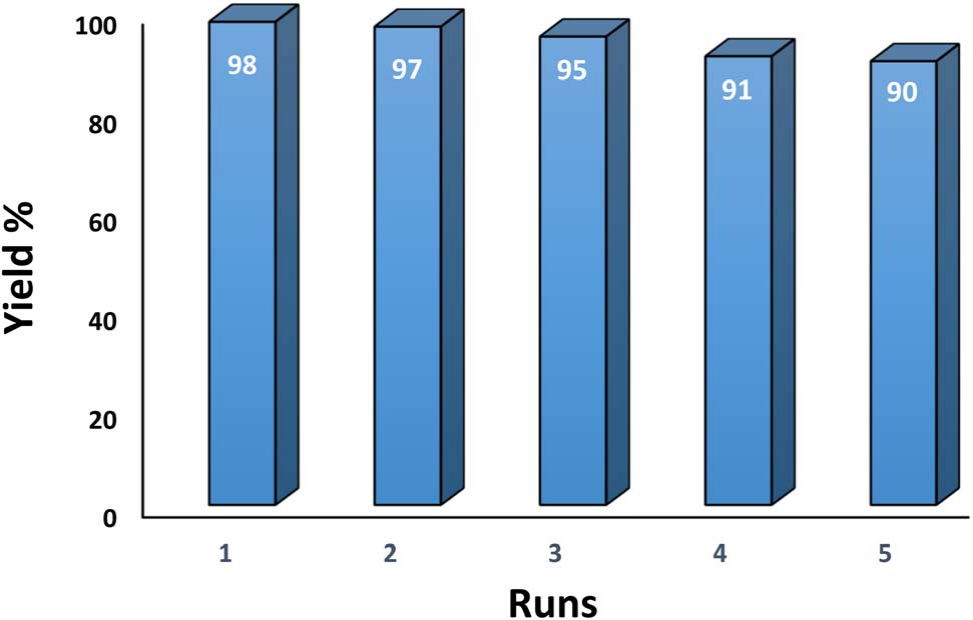
Recyclability of the SMNPs@DABCO@eosin nanophotocatalyst.

**Fig. 12:**
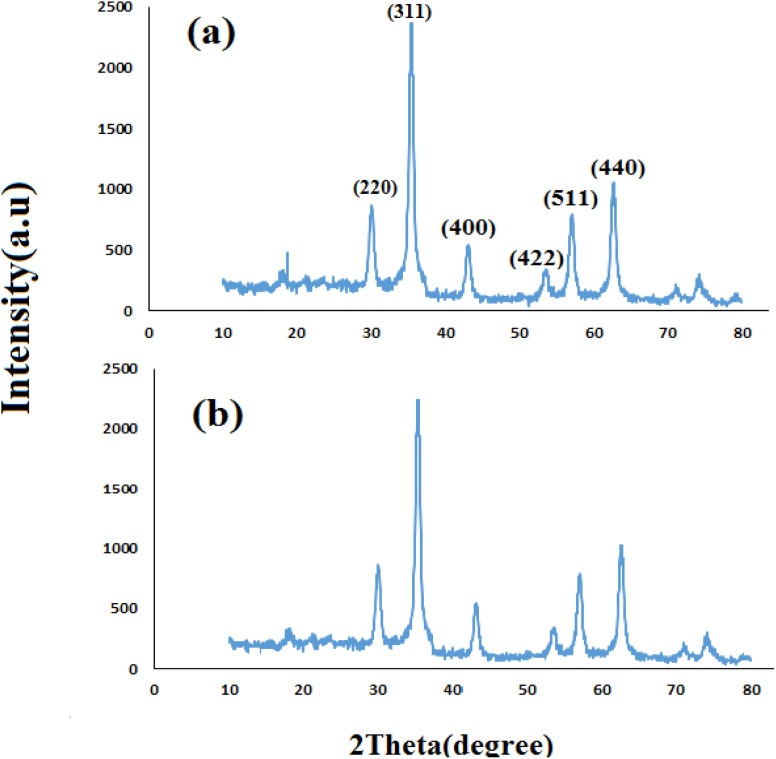
X-ray diffractions (XRDs) of the fresh (a) and reused (b) SMNP@DABCO@eosin nanophotocatalyst.

Reusability is one of the key parameters for evaluating the performance of catalysts. A catalyst is of great importance in the industry when it can be easily recovered and reused. Therefore to study the stability and reusability of nanoparticles of SMNPs@DABCO@eosin, after completion of the first run, toluene was appended and the catalyst was detached from the reaction mixture *via* a simple filtration. The filtered catalyst was washed with a copious amount of toluene, then dried at room temperature and reused in the same reaction for five consecutive runs (Fig. 11). It was found that SMNPs@DABCO@eosin could be reused five times without a significant decrease in extraction efficiency. The results proved acceptable reusability of the SMNPs@DABCO@eosin photocatalyst. XRD of the final reused nanocatalyst was compared with that of the fresh one. Interestingly, the nature and chemical composition of the reused nanomaterial after the fifth run was closely comparable with that of the fresh one. This study proved the stability of the nanophotocatalyst after at least five runs (Fig. 12).

### 3.3. Suggesting a plausible reaction pathway

**Fig. 13:**
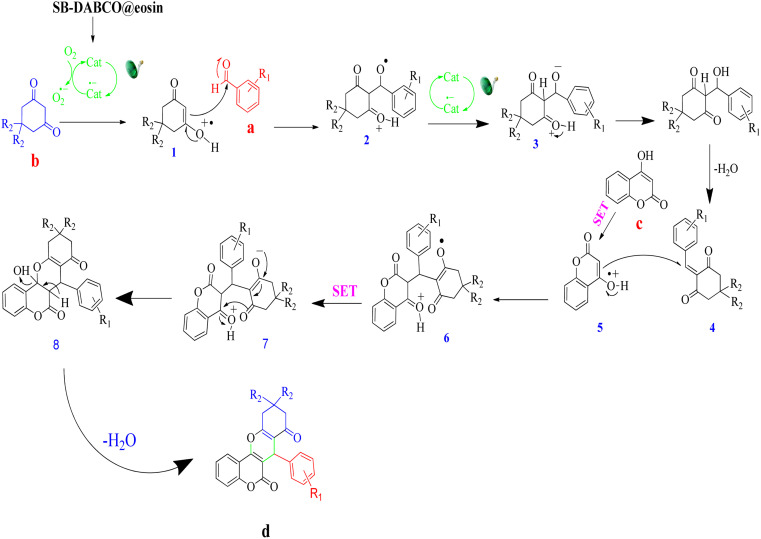
A probable reaction pathway for the condensation reaction.

A feasible mechanism for the generation of chromeno[4,3-*b*]chromene by SMNPs@DABCO@eosin is given in Fig. 13. It is proposed that the photoredox catalyst eosin Y is converted to excited state eosin Y* upon visible light irradiation, and then this eosin Y* undergoes reductive quenching by 1,3-dicarbonyl compound (b) to create the radical cation (1) and converts to the radical anion of eosin Y. Now this radical cation (1) is reacted with aldehyde (a) to form (2), and simultaneously eosin Y undergoes single electron transfer (SET) to produce compound (2) to (3). Dehydration of the compound (3) produces the β-dicarbonyl-enone (4) which can act as a Michael acceptor. Again 4-hydroxycoumarin (c) converts into a radical cation (5) by the photo-redox catalyst eosin Y *via* the single electron transfer (SET), and now this radical cation (5) undergoes Michael type addition reaction with β-dicarbonyl-enone (4) and furnishes a compound (6). At the same time, eosin Y undergoes single electron transfer (SET) to convert (6) into (7). Finally (7) on a simple dehydration reaction leads to the targeted molecule (d).^81^

## 4. Conclusions

In conclusion, we have successfully introduced an impressive route for the production of chromeno[4,3-*b*] chromene in the presence of a metal free catalyst under green LED irradiation. Eosin Y as a low cost and readily available photoredox catalyst has surely proven to be a superior green replacing reagent. We are very hopeful that this methodology will play a fundamental role in medicinal chemistry, natural product chemistry and organic synthesis by serving as a robust and economical alternative way to synthesize target molecules.

## Conflicts of interest

There are no conflicts to declare.

## Supplementary Material
